# Family Perspectives Related to Caring for Mental Health Care Users: A Case Study in the Long-Term Mental Health Institutions of Limpopo Province, South Africa

**DOI:** 10.3390/ijerph191710511

**Published:** 2022-08-24

**Authors:** Nkhensani F. Mabunda, Mutshinyalo L. Mangena-Netshikweta, Rachel T. Lebese, Foluke C. Olaniyi

**Affiliations:** 1 Department of Advanced Nursing Science, Faculty of Health Sciences, University of Venda, Thohoyandou 0950, South Africa; 2 Research Department, Faculty of Health Sciences, University of Venda, Thohoyandou 0950, South Africa; 3 Department of Public Health, Faculty of Health Sciences, University of Venda, Thohoyandou 0950, South Africa

**Keywords:** family perspectives, mental health care users, substance abuse, mental health stigmatization

## Abstract

Family involvement in long-term mental health care is a significant therapeutic aspect in managing mentally ill patients. This study aimed to determine the perspectives of family members about caring for mental health care users at selected long-term mental health institutions in Limpopo Province. A qualitative explorative and contextual descriptive design was used. Purposive sampling was used to select family members with mental health care users admitted in long-term health institutions in Limpopo Province. Data were collected with in-depth individual interviews aided by an audio recorder and field notes. Data were qualitatively analysed. Trustworthiness and ethical considerations were ensured. Two themes yielded from the interviews: Perspectives of family members about their involvement in the care of mental health care users and difficulties in caring for mental health care users at home when granted leave of absence or discharged. Sub-themes: Caring for mental health care users leads to an understanding of mental illness; Lack of skill and inability to monitor mental health care users at home; Mental health care users abuse substances during leave of absence which makes family reluctant to request them for visit; Caring for mental health care users at home viewed as a difficult task and stigma from the community. The challenges experienced by family members contribute to poor interaction with mentally ill patients. We recommend that family members of mental health care users be educated about mental illnesses and encouraged to participate in the care of the patients.

## 1. Introduction

To achieve the best psychiatric care, the World Psychiatric Association has recommended that acute or rehabilitation situations in psychiatric care be managed as a joint effort of mental health professionals, patients and caregivers (family members of mental health care users) [1]. Family involvement is a significant factor in the treatment and recovery of patients with mental illness, as mental health problems have been associated with an assortment of social and psychological processes in one’s family and family involvements have been reported to help with relapse prevention and reduced hospital stays for mental health users [1,2,3]. However, caring for mental health care users (MHCUs) is associated with great psychological and psychiatric morbidity for the family members themselves [4]. Families with MHCUs experience a range of negative emotional states that include irritation and frustration when they think of their loved ones with mental illness. Families may also experience varying levels of negative effects, such as stress, exhaustion, and physical health problems [5]. When family members refrain from caring for MHCUs because of these negative experiences, the recovery of MHCUs can be impeded [6].

Family members’ perspectives on caring for MHCUs is very important as reduced interaction with MHCUs by their family members can result in deterioration of health conditions and higher hospitalisation rates for MHCUs. MHCUs have been reported to experience feelings of isolation and loneliness when they are not visited while on admission in the hospitals; while visitations from their family members allay anxiety and provide feelings of worthiness. This shows that promoting family visits helped patients feel supported and connected to their family members [7]. Additionally, families’ involvement in MHCUs’ care create a trusting relationship and minimizes relapse. It also provides an atmosphere that nurtures equal partnership in mental health care services. Therefore, the involvement of family members in mental health care is often designated as a positive response that benefits MHCUs, family, and health professionals [8].

Family members of MHCUs have been reported to exhibit physical exhaustion, frustration, anger, fear, and other negative experiences when caring for mental health patients even after the patients have been stabilised in mental health hospitals [9,10,11]. Thus, it has been suggested that these families should be provided with sufficient information about coping with MHCUs when they are granted leave of absence (LOA) from mental health hospitals. LOA refers to the specified period of time when a patient is authorized to exit the hospital premises in the company of a staff, family or friend [12]. Understanding the importance of treatment compliance, early symptoms and signs of relapse, ways to deal with inexplicable and violent behaviours as well as various tactics for the management of MHCUs at home during LOA will empower the family members to better manage patients [13].

This study was conducted as a result of the odd behaviours observed among the family members of mentally ill patients admitted to long-term mental health institutions (MHI) in Limpopo Province, South Africa. Many of the MHCUs are not sufficiently visited by their family members and many family members seem to be unprepared to accept or stay with the MHCUs when discharged or granted leave of absence (LOA). In some instances where family members accept these patients during LOA, they are returned to the mental health institutions before the expected date, with complaints that the MHCUs refuse to take treatment at home, some abuse dagga and alcohol, while some are not accepted by community members because of their odd behaviours. This observation forms the basis of the research questions that this study aims to address: What are the perspectives of family members of mental health care users about their relatives who are suffering from mental illnesses? Additionally, what are their experiences while caring for these relatives at home during their LOA or after being discharged from MHIs?

Thus, this study seeks to describe the perspectives of family members regarding caring for MHCUs who are undergoing treatments in Limpopo Province mental health institutions so that they can be better equipped with relevant information and coping mechanisms, which could help them join hands with mental health care professionals to provide the optimal care for their MHCUs family members.

## 2. Materials and Methods

### 2.1. Research Design

A qualitative, exploratory, and descriptive research design was adopted in this study. This design was chosen because it helps the researcher gain an in-depth understanding of the phenomenon under consideration and as such, be able to describe in detail the perspectives of family members of MHCUs regarding their relatives with mental illness and their involvement in the care of the patients [14].

### 2.2. Study Setting

This study was conducted at selected MHIs at Mopani, Vhembe, and Capricorn Districts of the Limpopo Province. Limpopo Province has three long-term MHIs in the mentioned districts, in addition, all community hospitals in the province do have a psychiatric ward which houses acute psychiatric patients. Psychiatric services are also offered in primary health care setting including mobile clinics.

### 2.3. Population and Sampling

The population comprised of members of families who have MHCUs admitted at the selected MHIs. Purposive sampling was used to select participants who are at least 18 years of age, who have cared for the MHCUs closely within the same household during their LOA or after being discharged from the hospitals and who volunteered to participate in this study. Family members who were minors (younger than 18 years old) were excluded because they could not directly give consent to participate in the study and those who did not share the same household with the MHCUs were also excluded because they would not be able to provide full details of the MHCUs behaviours during their LOA.

### 2.4. Data Collection Method

Unstructured in-depth interviews were conducted with 21 family members caring for MHCUs in their homes who were on LOA as at the time of the data collection for this study. The participants were recruited through the institutions’ social workers who recruited the families on behalf of the researcher and contacted them telephonically to secure an appointment. The researcher visited each of the families on their respective appointment dates, provided a detailed explanation to the prospective participants about the nature and the purpose of this study, and then obtained their consent in writing before they were interviewed. The interview was directed by one central question which was translated into three main languages (Xitsonga, Tshivenda, and Sotho) being spoken by the people in the community: “Can you explain to me what it feels like to have a mental health care user in your family?”. The question was translated to ensure that the participants feel at ease when expressing themselves and the translation was performed by language experts from the language department at the University of Venda.

Xitsonga: mi nga ni hlamusela leswaku mi ti twa njhani kuva mi ri na muvabyi wa miehleketo laha muntini,

Tshivenda: vha nga ntalutshedza uri vha di pfa hani musi vhana mulwadze wa muhumbulo hafha hayani,

Sotho: le ka nhlalosetsa gore le ikwa jwang goba le molwetsi wa go lwala kgopolong ka mo lapeng.

This central question was followed-up by probing questions based on the responses of the participants, to be able to obtain more detailed information about their experiences [15,16]. The instrument (interview guide) was pre-tested before being used in the main study.

### 2.5. Data Analysis

Tech’s Technique was used to analyse the data [17] and reduce data to an understandable, interpretable form [18] as well as to organize and provide structure [14]. Verbatim transcripts were made by the researcher by listening to the audio recordings before data analysis was conducted. The researcher requested the assistance of an independent coder, who was familiar with the Tesch’s open coding, to confirm the developed themes [17,19].

### 2.6. Ethical Consideration

Ethical clearance was granted by the Research Ethics Committee of the University of Venda (SHS/16/PDC/35/1611), and permission to conduct the study was obtained from the Department of Health, Limpopo Province (LP/4/2/2), as well as from Mopani, Vhembe, and Capricorn Districts. Ethical principles such as informed consent, the right to privacy, confidentiality, autonomy, and voluntary participation were duly adhered to.

## 3. Results

### 3.1. Demographic Data

Table 1 displays the demographic characteristics of the participants. The sample is comprised of more employed (*n* = 12) family members than unemployed (*n* = 9), and the majority (*n* = 13) were females.

### 3.2. Themes and Sub-Themes from Interviews

The following themes and sub-themes emerged from the in-depth interviews conducted with family members of MHCUs, with regard to their perspectives related to caring for MHCUs admitted to a long-term MHI (Table 2).

#### 3.2.1. Theme One: Perspectives of Family Members about Their Involvement in the Care of MHCUs

Participants revealed that they were ready to be involved in the care of their family members with mental illnesses. They outlined the benefits associated with such involvement, especially an increase in their knowledge and understanding of mental illnesses and how to manage them. However, they also indicated some challenges they faced while trying to involve themselves in the care of their family members with mental illnesses. The following sub-themes emerged from theme 1: (1) having MHCUs leads to an understanding of the mental illness; (2) lack of skills and inability to monitor MHCUs at home; and (3) substance abuse by MHCUs during LOA makes families reluctant to request that they visit home.

##### Sub-Theme One: Having MHCUs Leads to an Understanding of the Mental Illness

Some participants stated that they were aware that being involved in caring for MHCUs helps them better understand mental illnesses and how they are managed:

*It helps us as a family to understand how my younger brother is getting help in the hospital. It also helps us to understand why my younger brother is mentally ill.* (Participant 1).

*Being involved in caring for our family members helps us to understand the causes of mental illness. We thought that my child was bewitched while studying at Technical College... The nurses even educate us on how to assist him regarding taking treatment.* (Participant 3).

*Being involved in mental health care helps us to understand mental illness, the way our child is going to be assisted and also to continue with treatment at home. We are unable to manage our child without the relevant information.* (Participant 6).

*It helps me to understand and follow-up on my child’s illness... I am ready to supervise him on treatment compliance.* (Participant 5).

The statements above clarify that families with MHCUs benefit when involved in mental health care by having a better understanding on mental illnesses and the treatment modalities. The relevant information provided by the healthcare team, especially the nurses, help the families better manage their relative with mental illnesses.

##### Sub-Theme Two: Lack of Skills and Inability to Monitor MHCUs at Home

Participants also indicated some challenges they face while trying to involved themselves in the care of their family members with mental illnesses. These included a lack of the relevant skills by the family members to monitor MHCUs at home as well as the unwillingness of the MHCUs to spend time with the family or receive care from them. Participants also indicated that they were unable to convince MHCUs to do follow-ups at the local clinic to prevent the consequences of a relapse:

*The problem is that I am unable to manage him at home. He says that we should not take part in monitoring treatment compliance; we are not nurses.* (Participant 8).

*I even asked the social worker to do home visit together with my uncle (the MHCU). They came together with my uncle. We prepare food to serve them. My uncle asked the social worker to hurry saying that they should depart because they can miss hospital lunch.* (Participant 13).

*My brother refuses to do follow-ups at clinic for the treatment while at home, saying that he will go back next week, until he relapses. The problem is that he does not want his treatment to be supervised.* (Participant 1).

These responses raise a concern regarding the lack of necessary skills by family members to monitor MHCUs at home when discharged or granted LOA. Thus, families with MHCUs are unable to enforce treatment compliance for their loved ones with mental illnesses.

##### Sub-Theme Three: Substance Abuse by MHCUs during LOA Makes Families Reluctant to Request That They Visit Home

Some participants explained that MHCUs abuse substances, an act which discourages their families from visiting them or requesting that they visit during LOA:

*We are unable to pay visit; he begs us to bring some dagga when visiting the hospital, saying that he don’t like cool drink and snakes that we are bringing at the hospital. We think it does not help us to visit him at the hospital because he became angry, saying we should try by all means to hide the dagga.* (Participant 12).

*When granted LOA or discharged, he mixes the treatment with dagga. We are aware that our child abuses alcohol, he even knows that smoking dagga can affect his grant that he is receiving.* (Participant 10).

Substance abuse is a very common comorbid health problem with mental illnesses worldwide. MHCUs who are addicted to substances tend to abuse the same substances when granted LOA or discharged and as such discourage their family members to continue to care for them at home.

#### 3.2.2. Theme Two: Difficulties in Caring for MHCUs at Home When Granted Leave of Absence or Discharged

Participants revealed that caring for MHCUs at home during their LOA was problematic, due to MHCUs’ odd behaviours while at home, and stigmatization from community members. Thus, the families sometimes end up returning the MHCUs back to the hospitals before the expected date of return. The following sub-themes were identified: (1) caring for MHCUs at home viewed as a difficult task; and (2) stigma from community members.

##### Sub-Theme One: Caring for MHCUs at Home Viewed as a Difficult Task

Participants revealed that caring for MHCUs at home during their LOA was problematic, especially when the carer is unemployed. Additionally, families seem to be discouraged by MHCUs’ odd behaviours (such as not maintaining personal hygiene or causing troubles) while at home.

*I won’t take him for LOA. He does not have time to bath, he roams around the village aimlessly being dirty. Police say that they are tired to be called to take my uncle back to the hospital. He is dangerous to the community and even to the family.* (Participant 2).

*His mental illness affected the whole family. I do not have money, I am not working. I am depending on my mother’s grant. That is why we are not visiting him or asking for him to visit.* (Participant 15).

*I am paying R300.00 every month for the neighbor’s house that he burned of which it will take long to finish paying in instalments (looks sad). I think it is a punishment to have mental illness within the family. I don’t think I can take him again. I am afraid that he can do the same thing that can leave me paying another money.* (Participant 8).

Family members with MHCUs also lack continuous support from South African Police Services when MHCUs relapse. As a result, caring for MHCUs at home exposes the family members to remarkable distress, heartache, and day-to-day stress, which can result in significant risks to their health and wellbeing.

##### Sub-Theme Two: Stigma from Community Members

Some participants claimed that they were discriminated against by community members for having mental illness within the family. They also complained that they were usually accused of having done something wrong and were being punished by having mentally ill child or relative. This stigma from community members was problematic.

*My neighbors, colleagues and even the community are not happy when my child is on LOA. They discriminate against me saying that there is something wrong which I did that is why I have a mentally ill child. I used to lock the gate, so that my child could not go out. It does not help because they also laugh when they see him roaming in the yard. I am really depressed.* (Participant 17).

*I went to sangoma (traditional healers) to try to get help about my brothers’ mental illness as he is no longer wanted by the community members. The community members even complained to the “induna”. That is why he came with the letter to indicate that he is no longer wanted at our village (looks sad).* (Participant 21).

These statements raise a concern that family members with MHCUs are exposed to stigma when they are involved in the care of their relatives with mental illnesses.

## 4. Discussion

Leave of absence (LOA) was identified as an effective therapeutic milieu in recovery-oriented and quality-of-life value for MHCUs admitted in long-term mental health institutions. This period allows them to relate more intimately with their families and communities and it has been found to be significant in minimising the risk of abscond from the hospital [12]. During this period, the management of MHCUs is largely taken over by the family members. Family support has been viewed as a crucial factor in recovery from mental illnesses [13].

In this study, most family members of MHCUs were ready to be involved in their mental health care. However, such involvement is burdensome for many reasons which were highlighted by the participants. When family members show their willingness to participate in the care of their mentally ill relatives, they are able to obtain the relevant information and assistance from the healthcare team involved in the management of the patients, and are thus able to identify the risk factors and causes of mental disorders, become familiar with the treatment of the disorders, as well as experience the satisfaction and positive feelings of caregiving [9].

To achieve a good result of optimal mental health care during LOA, family members of MHCUs need to be supported and armed with the necessary information and skills needed to take care of their relatives. While most of the information can be obtained from nurses as mentioned by some participants in this study, it has also been suggested that such family members participate in support groups with similar families where they can share their experiences, challenges and suggest coping mechanisms for one another [20].

Another challenge to family involvement in MHCUs care as encountered in this study is substance use by the patients. Substance use by MHCUs has been implicated in the disruption of social support from the family and community as well as relapse, resulting in MHCUs’ readmission to the hospital even before the completion of the scheduled dates for the LOA [21]. Family members are reluctant to request LOAs for MHCUs who abuse substances during this period, because substance abuse makes it more difficult for families to cope with caring for MHCUs at home [22].

Family members often view caring for MHCUs at home as a difficult task because they also have to purse wellness, a physical and psychological strength related to wellbeing and life satisfaction for themselves and MHCUs usually constitute extra burdens. When there are other psychological problems in a family, it becomes more difficult to care for a MHCU [23,24]. In addition, the family members of MHCUs experience stigma from community members as an obstacle to access social and formal support for themselves and their relative with mental illness [25].

Support from the community has been linked to better outcomes in mental illnesses’ recovery and rehabilitation [26]. The World Health Organization also recommends comprehensive, integrated mental health care which involves promotion and prevention programmes in the community, as a means of combating the problem of mental illnesses [27]. The community is expected to be involved in the prevention and management of mental illnesses through the provision of psychosocial support, the formation of support groups, creation of awareness on substance abuse and supporting community-based rehabilitation programs [27]. However, a community that stigmatizes MHCUs and their family members cannot perform these roles. Social exclusion and reduced contact with members of the community have been implicated in the exacerbation of mental illnesses [26].

## 5. Conclusions

Negative attitudes of family members towards MHCUs contribute to feeling of rejection. When families are involved in treatment plan, both family and MHCUs will benefit; hence, the quality of mental health services might improve. We recommend that families of MHCUs be encouraged and equipped with the necessary skills to participate in their care. Additionally, the leaders of the communities where MHCUs come from should be educated on the needs of community support whenever the patients are on LOA and when they are eventually discharged from MHIs.

## Figures and Tables

**Table 1:** Demographic characteristics of participants.

Participants	Age Group (in Years)	Employment Status				Sub-Total
		Employed		Unemployed		
Gender		Male	Female	Male	Female	
	20–30	1	-	1	1	3
	31–40	1	3	1	3	8
	41–50	2	4	1	2	9
	>50	1	-	-	-	1
	Total	5	7	3	6	21

**Table 2:** Themes and sub-themes emerging from interviews.

Themes	Sub-Themes
1. Perspectives of family members about their involvement in the care of MHCUs	1.1 Having MHCUs leads to understanding of the mental illness 1.2 Lack of skills and inability to monitor MHCUs at home 1.3 MHCUs abuse substances during LOA, which makes families reluctant to request them to visit home
2. Difficulties in caring for MHCUs at home when granted leave of absence or discharged	2.1 Caring for MHCUs at home viewed as a difficult task 2.2 Stigma from community members

## Data Availability

Data analysed in this study can be obtained from the first author upon reasonable request.
